# Complications after prone PCNL in pediatric, adult and geriatric patients – a single center experience over 7 years

**DOI:** 10.1590/S1677-5538.IBJU.2016.0563

**Published:** 2017

**Authors:** Sumit Kumar, Ramaiah Keshavamurthy, Vilvapathy Senguttuvan Karthikeyan, Ashwin Mallya

**Affiliations:** 1 Department of Urology, Institute of Nephrourology, Bangalore, India

**Keywords:** Kidney Calculi, Nephrostomy, Percutaneous, complications [Subheading]

## Abstract

**Introduction:**

CROES-Clavien system (CCS) for grading complications in percutaneous nephrolithotomy (PCNL) is a step towards standardization of outcomes. We categorized complications based on CCS and predicted risk factors across the entire cohort and individually for pediatric (P: ≤18 years), adult (A: 19-65 years) and geriatric (G: ≥65 years) subgroups to assess the risk factors in each subset. We assessed association of complications with length of hospitalization (LOH) and operation time (OT).

**Materials and Methods:**

Retrospective record review of unilateral PCNL performed between January 2009-September 2015 at a tertiary care center in India, performing around 150 PCNL per year.

**Results:**

Out of 922 (P=61; A=794; G=67) PCNL, 259 (28.09%) complications occurred with CCS I, II, III and IV constituting 152 (16.49%), 72 (7.81%), 31 (3.36%) and 4 (0.43%) respectively and its distribution was similar across the subsets and majority (224; 24.3%) were minor (CCS-1, 2). Placement of a nephrostomy (47.4%; 18/38) in Group P, supracostal access, ≥2 punctures, higher GSS, nephrostomy, staghorn stones, ≥2 stones, stone size in Group A and hydronephrosis and prolonged OT in Group G were significantly associated with complications. On logistic regression, need of nephrostomy (adj. OR - 4.549), OT (adj. OR - 1.364) and supracostal access (adj. OR - 1.471) significantly contributed to complications in the study population. LOH was found to be significantly associated with complications (p<0.001).

**Conclusions:**

Contrary to the belief that extremes of ages are associated with complications of prone PCNL, we found age does not alter the incidence or grade of complications and LOH.

## INTRODUCTION

Percutaneous nephrolithotomy (PCNL) is the standard of care for large and complex renal stones across all age Groups (1, 2). Though it is highly efficacious, it is associated with morbidity. Reported complication rates following PCNL range up to 83% and there is marked heterogeneity in reporting post-PCNL complications with underreporting of minor complications (3, 4). The modified Clavien-Dindo grading system for complications in urology has now been adapted for PCNL by the Clinical Research Office of the Endourological Society (CROES) Study Group, referred to as CROES-Clavien score (CCS) (4, 5). This score has been successfully used to report complications after pediatric PCNL (5).

Prone PCNL is the standard at most centres (6). Supine PCNL, introduced by Valdivia in late 1980s, is being adopted by experts due to the proposed advantages which actually are the limitations of prone PCNL. These include anesthetic benefits like decreased cardiovascular and respiratory distress. From the endourologist’s point of view, a more horizontal sheath position in supine PCNL improves the chances of spontaneous stone expulsion during the procedure and keeps the operator’s hands away from the field of radiation (6, 7). Two recent meta-analyses showed that supine PCNL have similar LOH and complications but a significantly lesser stone free rates when compared to prone PCNL (8, 9). We routinely perform prone PCNL at our institute. We report the complications of prone PCNL in the entire cohort of PCNL patients using the CCS. We subdivided them into pediatric, adult and geriatric subgroups to assess the risk factors in each subset. We also intended to assess the association of complications with length of hospitalization (LOH) and operation time (OT).

## MATERALS AND METHODS

This is a retrospective record review of unilateral PCNL performed between January 2009 and September 2015 at a tertiary care center in South India, performing around 150 PCNL per year. Institutional research review board approval was obtained.

### Data collection

Being a teaching institute, all case records were prospectively filled in by resident urology trainees and these were checked by consultants. These data were then entered in Microsoft Excel 2010 spreadsheet by the authors. After checking for the accuracy of the data, it was transferred to SPSS version 20 (IBM Corp., Armonk, NY, USA) for statistical analysis.

### Evaluation

Preoperative evaluation included hemoglobin, serum creatinine, serum electrolytes, urine analysis, urine culture and ultrasound of kidneys, ureter and bladder region (KUB), plain X-ray of KUB region (XRKUB) and intravenous urography or computed tomography KUB region. XRKUB and renal ultrasound were performed 48 hours after PCNL. Stone free status (SFS) was defined as absence of any residual stone >4mm on XRKUB (10).

### Perioperative data

Patient factors recorded included age, gender, Charlson comorbidity index (CCI) and past surgical history for stone disease. Stone characteristics included stone size (mm), location, stone count, staghorn stones and Hounsfield units. Intraoperative data collected were supracostal/infracostal entry, calyces punctured, number of access tracts, tract size, OT and postoperative drainage. Postoperative outcomes assessed were SFS, residual stone, complications based on CCS, LOH and analgesic requirements. CCS grades 1 and 2 constitute minor complications and grades 3 and 4 have been grouped as major complications (4, 5). We also analyzed the outcomes based on OT cut-off of 75 minutes as defined by Smith et al. (10).

We used the Guy stone score as proposed by Thomas et al to predict the outcomes of PCNL (11). It comprises of 4 grades - grade I includes solitary renal calculus in mid/lower pole or solitary calculus in the renal pelvis with simple anatomy; grade II includes solitary calculus in the upper pole or multiple stones in a patient with simple anatomy or a solitary calculus in a patient with abnormal anatomy; grade III includes multiple calculi in a patient with abnormal anatomy, calyceal diverticular or partial staghorn calculi and grade IV includes staghorn calculus or any renal calculus in patients with spina bifida or spinal cord injury (11).

## Surgical Technique

The procedure was performed under general anesthesia. In lithotomy position, an open-ended 5F/70cm ureteral catheter was placed on the side of stone through rigid cystoscope (Karl Storz Endoscopy, Tuttlingen, Germany). Patient was placed prone and retrograde pyelography was performed. Using the bull’s eye technique, appropriate calyx was punctured by the urologist with an 18G/15cm diamond-shaped trocar needle under fluoroscopy. A 0.032” hydrophilic guidewire was inserted and tract dilation was done up to the desired size. The maximum tract size was up to 30F and a 17F, 22F or 26F nephroscope (Karl Storz Endoscopy, Tuttlingen, Germany) was used. Lithotripsy was done by pneumatic lithotripter (Nidhi Lith Digi, Nidhi Meditech Systems, India). Postoperative drainage was based on intraoperative factors and surgeon choice. Ureteric catheter and nephrostomy tube were removed after 48 hours if the patient had no hematuria or fever. In case of hematuria or fever, these were removed 24 hours after hematuria and fever subsided. In patients with ureteric stents in situ, these were removed 4 weeks later.

## Statistical analysis

Data was tabulated and statistical analysis was performed using SPSS version 20. Continuous variables were depicted as mean with standard deviation and categorical variables as median and interquartile range. For tabulation and analysis, the study population was divided into three subgroups - pediatric (Group P -≤18 years), adults (Group A - 19 - 65 years) and geriatric (Group G -≥65 years) (12, 13). Student t test (two tailed, independent) and one way ANOVA or Mann Whitney U test and Kruskal Wallis test as appropriate were used for continuous variables based on the normality of the distribution. Chi-square and Fisher exact test were used to compare parameters on categorical scale. Binomial logistic regression analysis was used to identify independent predictors for complications. A p value of <0.05 was considered statistically significant.

## RESULTS

A total of 922 patients were eligible for analysis. Group P had 61 (6.62%) patients with a mean (±SD) age of 12.6±4.8 years, Group A had 794 (86.12%) patients with a mean age of 40.9±11.5 years and Group G had 67 (7.27%) patients with a mean age of 68.8±4.9 years. The mean (±SD) stone size was lower in Group P (19.3±7.1mm) and similar in Groups A (22.4±7.6mm) and G (22.7±8.0mm). Patient, stone characteristics and OT are depicted in Table-1. Across the three Groups, distribution of stone location, presence of staghorn stones, hydronephrosis, anomalous kidneys, side of stone and GSS were similar. A total of 259 (28.09%) complications occurred in the study cohort with CCS I, II, III and IV constituting 152 (16.49%), 72 (7.81%), 31 (3.36%) and 4 (0.43%) respectively. Majority of the complications were minor (224; 24.30%). Proportion of complications based on CCS was similar across all three age groups (Table-2).

**Table 1:** Patient, stone characteristics and OT based on age groups.

Characteristics	Pediatric	Adult	Geriatric	P value
**Age, years**				<0.001
Mean±SD	12.56±4.8	40.91±11.5	68.78 ±4.9	
**Stone size, mm**				0.008
Mean±SD	19.31±7.13	22.41±7.59	22.69±7.98	
**Staghorn stones**				0.064
N(%)	7 (11.5)	99 (12.5)	15 (22.4)	
**GSS N(%)**				
I	39 (63.9)	467 (58.9)	39 (58.2)	0.414
II	12 (19.7)	164 (20.7)	11 (16.4)	
III	3 (4.9)	64 (8.1)	3 (4.5)	
IV	7 (11.5)	98 (12.4)	14 (20.9)	
**CCI N(%)**				
≤2	61 (100)	746 (94)	27 (40.3)	<0.001
>2	0	48 (6)	40 (59.7)	
**OT, min**				
Mean±SD	81.48±30.71	78.17±25.52	77.09±24.64	0.58

**Table 2:** Clavien-CROES complications – overall and based on age groups.

CCS grade	CCS grade	Description	N (%)	Pediatric	Adult	Geriatric	P value
**Minor**	**I**	Postoperative fever (38°C) without change of antibiotics	78 (8.5)	18 (85.7)	193 (86.6)	13 (86.7)	0.691
		Bleeding without need for blood transfusion or required single episode of nephrostomy clamping or skin compression/ pressure dressing	54 (5.9)				
		Urine leakage - watchful waiting	20 (2.2)				
		Renal pelvic perforation - watchful waiting	5 (0.5)				
		Intestinal obstruction - without nasogastric decompression	2 (0.2)				
		Subcapsular hematoma - watchful waiting	1 (0.1)				
	**II**	Bleeding requiring blood transfusion	36 (3.9)				
		Postoperative fever (>38°C) with change of antibiotics	28 (3)				
		Postoperative ileus - nasogastric decompression	4 (0.4)				
		Pulmonary edema – diuretics	3 (0.3)				
**Major**	**IIIA**	Ureteric stent without general anesthesia	13 (1.4)	3 (14.3)	30 (13.4)	2 (13.3)	
		Renal pelvic perforation - prolonged nephrostomy tube or postoperative ureteric stenting without general anesthesia	9 (1)				
		Hydrothorax managed by intercostal drainage under local anesthesia	9 (1)				
	**IVA**	Urosepsis - ICU management	2 (0.2)				
		Acute renal failure - ICU management	1(0.1)				
		Heart failure -ICU management	1 (0.1)				

### Predictors of complications - overall

On univariate analysis, the factors contributing to complications included supracostal access (p=0.001), ≥2 punctures (p=0.002), nephrostomy insertion (p <0.001), increasing GSS class (p=0.042), increasing stone size (p=0.011), presence of staghorn calculi (p=0.008), multiple stones (p=0.022) and prolonged OT (p <0.001). On logistic regression, need of nephrostomy (adj. OR - 4.549), OT (adj. OR - 1.364) and supracostal access (adj. OR - 1.471) significantly contributed to complications (Table-3). When OT exceeded 75 minutes, a significantly higher proportion (36.5%; 134/367) of patients developed complications when compared to 23.2% (129/555) developing complications when OT was <75 min (p <0.0001).

**Table 3:** Logistic regression for predictors of complications.

Predictors	Adjusted OR	95% CI for Adjusted OR		P value

		Lower	Upper	
Supracostal	1.471	1.070	2.023	0.017
More than one puncture	1.283	.791	2.080	0.312
Nephrostomy	4.549	2.991	6.917	<0.001
Staghorn calculus	1.386	0.820	2.344	0.223
Stone size in mm	0.996	0.971	1.022	0.777
Duration of procedure >75min	1.364	0.986	1.887	0.048
**Age group**				
Pediatric	0.244	-	-	-
Adult	0.653	0.361	1.181	0.159
Geriatric	0.510	0.226	1.150	0.105
Stone in more than one location	0.874	0.586	1.305	0.511

### Outcomes – overall

A stone free rate of 78.31% (722/922) was achieved. Out of the remaining 200 patients (21.7%) with residual calculus, relook PCNL was performed for 78 (8.46%) patients and 58 (6.29%) required shock wave lithotripsy (SWL) for stone clearance. The mean (±SD) LOH was 4.56±3.15 days (Table-4). The mean LOH was higher (6.61±3.97 days) in patients with complications (vs. 3.74±2.29 days without complications; p <0.001). LOH increased proportionately with increasing CCS. The proportion of patients having prolonged LOH >3 days [CCS 1 - 120 (55.6%); 2 - 64 (29.6%); 3 - 27 (12.6%); 4 - 1.9%] was higher as CCS increased when compared to LOH ≤3 days [CCS 1 - 29 (67.4%); CCS 2 - 7 (16.3%); 3 - 4 (9.3%); 4 - nil]. However, it was not statistically insignificant (p=0.202).

**Table 4:** Outcomes – overall and among three age groups.

Characteristics	Pediatric	Adult	Geriatric	P value	Overall
**Stone free status**					**722 (78.3)**
N (%)	50 (82)	623 (78.4)	49 (73.1)	0.718	
**Residual stone**					**200 (21.7)**
N (%)	11 (18)	171 (21.5)	18 (26.9)	0.713	
**Complications**					**259 (28.1)**
N (%)	21 (34.4)	221(28)	17 (25.4)	0.484	
**Doses of analgesic**					**7.29±3.02**
Mean±SD	6.67±3.19	7.3±2.98	7.72±3.35	0.144	
**Relook PCNL**					**78 (8.5)**
N (%)	3 (4.9)	69 (8.7)	6 (9)	0.588	
**Length of hospitalization**					**4.56±3.15**
Mean±SD	4.3±3.08	4.55±3.18	4.93±2.81	0.512	

The mean (±SD) number of analgesic demands after PCNL was 7.29±3.02 and mean analgesic requirements were similar across all three age groups (Table-4). Proportion of minor and major complications based on CCS was similar with respect to increasing GSS and OT and placement of a nephrostomy. The distribution of complications based on CCS was similar across the three age groups (Table-5).

**Table 5:** Complications based on CROES-Clavien score across different age groups.

Age group	CROES-Clavien score-1	CROES-Clavien score-2	CROES-Clavien score-3	CROES-Clavien score-4	P value

	N (%)	N (%)	N (%)	N (%)	
**Pediatric**	13(61.9)	4 (19)	3 (14.3)	0	0.691
**Adult**	130(58.8)	60 (27.1)	26 (11.8)	4 (1.8)	
**Geriatric**	6 (35.3)	7 (41.2)	2 (11.8)	0	

### Predictors of complications – Pediatric subgroup

In Group P, 21 (21/61; 34.4%) patients had complications with minor complications contributing to 85.7% (18/21). Placement of a nephrostomy after PCNL (47.4%; 18/38) was significantly associated with complications while only 13% (3/23) without nephrostomy developed complications (p=0.006). The mean (±SD) OT was 81.48 (30.71) min. In patients with OT >75 min, 40.7% (11/27) patients developed complications while only 29.4% (10/34) of patients with OT <75 min developed complications (p=0.355).

### Predictors of complications – Adult subgroup

In adults, 224 (28.2%) patients had complications and 86.6% (194/224) of them were minor. Supracostal access (34.7% vs. infracostal access 23.7%; p=0.001), ≥2 punctures (40.7% vs. single 26.4%; p=0.005), higher GSS (I - 25.8%; II - 26.8%; III - 28.6%; IV - 40.2%; p=0.037), insertion of nephrostomy tube (36.8% vs. 11.3% without nephrostomy tube; p <0.001), presence of staghorn stones (39.8% vs. 26.3% without staghorn stones; p=0.005), stone count ≥2 (32.53% vs. 25.3% for single stones; p=0.013), stone size (23.59±8.11; p=0.007) and OT (85.11±27.75 min vs. 75.41±24.09 min in patients without complications; p <0.0001) had a significantly higher incidence of complications. With supracostal puncture, the proportion of severe complications increased (p <0.0001). In patients with OT >75 min, a significantly higher (36%; 113/314) proportion of patients developed complications while only 22.7% (108/476) of patients with OT <75 min developed complications (p <0.0001).

### Predictors of complications – Geriatric subgroup

Among geriatric patients, 15 (15/67; 22.4%) had complications with Clavien I and II constituting 86.7% (13/15) of them. Presence of hydronephrosis (58.3% vs. 18.2% without hydronephrosis; p=0.008) and prolonged OT (88.82±31.35 min vs. 73.10±20.80 min in patients without complications; p <0.0001) were significant predictors of complications. The mean (±SD) OT was 77.09 (24.64) min. Patients with supracostal access had increasing CCS grade of complications, however it was not statistically significant (p=0.064). In patients with OT >75 min a higher (38.5%; 10/26) proportion of patients developed complications while only 17.1% (7/41) of patients with OT <75 min developed complications (p=0.082).

## DISCUSSION

Complication rates for PCNL range from 20 - 83% (14). There is a need for standardized reporting of complication after PCNL (15). European Association of Urology (EAU) guidelines panel in 2012 highly recommended the use of CCS grading system as a uniform and standardized system to classify complications after PCNL. CCS is described based on the management of a given complication and it is not influenced by the potential risk to which a patient is exposed due to complication. Minor (grades 1 and 2) complications account for a high proportion and underreporting of low grade complications is not unusual (4).

Seitz et al. reported in their systemic review on complications of PCNL that fever is a common complication, with an overall incidence of 10.8% (16). The amount of irrigation fluid and operation duration influence postoperative infection. We observed fever as the most common complication in our study (106; 11.5%). It was graded as I in 8.46% (78) patients that could be managed without a change in antibiotics and as grade II in 3.04% (28) patients requiring a change in antibiotics based on urine culture and sensitivity report. Individual hospital protocol for perioperative antibiotic use could differ.

The second most common complication in our study was bleeding accounting for 9.76% (90) of patients. It was categorized as grade I in 5.86% (54) patients where bleeding was controlled by single episode of nephrostomy clamping, skin compression or pressure dressing and as grade II in 3.91% (36) patients who required blood transfusion. Seitz et al. reported that blood transfusion is required in 0 - 20% patients with an overall incidence of 7% (16.).

Ileus (0.22%, 2 patients), subcapsular hematoma (0.11%, 1 patient), urine leakage (2.17%, 20 patients) and renal pelvic perforation (0.54%, 5 patients) managed by watchful waiting needing no active intervention constituted grade I CCS complications. Ileus requiring nasogastric decompression (0.43%, 4 patients) and pulmonary edema requiring diuretics (0.33%, 3 patients) constituted grade II CCS complications.

Urine leakage (13 patients; 1.41%) and renal pelvic perforation (9 patients; 0.98%) managed by placement of nephrostomy tube or ureteric stents without general anesthesia were categorized as grade III-A. Hydrothorax (9 patients; 0.98%) managed by intercostal tube drainage under local anesthesia were also included in this Group. Lojanapiwat et al. reported 15.3% (26 patients) developed hydrothorax via supracostal puncture with only 5.3% (9 patients) requiring intercostal drainage (17). Patients requiring ICU care for urosepsis without multiorgan failure (2 patients; 0.22%), acute renal failure (1 patient; 0.11%) and heart failure (1 patient; 0.11%) were categorized as grade IV-A. None of our patients had grade III-B or IV-B complications.

De la Rosette et al. validated the modified Clavien-Dindo system for Urology on 5803 patients from 98 centers in 26 countries and proposed the CCS (4). In our study, complications based on CCS were observed in 28.1% patients. CCS I, II, IIIA and IVA constituted 152 (16.49%), 72 (7.81%), 31 (3.36%) and 4 (0.43%) patients respectively. The CROES global study Group reported an overall complication rate of 21.5% with low grade complications (grade I and II) accounting for 16.4%, grade III-a and III-b complications in 3.6% and grade IV in 0.5% patients (3).

Lee et al. reported that 15.4% of children and 17.9% of adults developed complications following PCNL (18). We observed that the distribution of complications across the pediatric, adult and geriatric cohorts are similar with minor complications accounting for 85.7%, 86.6% and 86.7%% respectively. Sahin et al. retrospectively compared the outcomes of 27 elderly patients of PCNL with 166 younger patients and reported similar stone-free rate, complications and length of stay between them (19).

Goyal et al. reported that OT was the only independent predictor of complications (5). In our study, we observed that in the pediatric subset, nephrostomy tube insertion was the only significantly predictor of complications. Though OT was higher in patients with complications, it could not achieve statistical significance. We noted that supracostal access, presence of nephrostomy tube and duration of procedure were the only significant predictors on multivariate analysis in the study population and adult cohorts. In the geriatric population also, OT was found as the only significant predictor of complication.

We further categorized our patients based on OT cut off of 75 min as proposed by de la Rosette et al. and found that the complication rates increased significantly in these patients (4). We also observed that the likelihood of hospital stay increased with increasing severity of complications. de la Rosette et al. used postoperative LOH as a surrogate measure for the severity of complications and showed that OT >75 min increased complications and also prolonged LOH (4). This could prove useful in counseling patients undergoing PCNL with an increased chance of complications. Prolonged OT and placement of nephrostomy may also indicate the greater level of difficulty for PCNL.

Our study has few merits. We have categorized complications based on CCS which was proposed as a step towards standardization of reporting of complications in PCNL. We noted that adults contributed to the majority of our patients and thus the overall outcomes were similar to that in adult subgroup. Most published studies report complications as a whole and do not sub-classify based on age and there is a dearth of reports on complications in pediatric and geriatric PCNL. To the best of our knowledge, ours is the first study stratifying patients into subgroups based on age. Though there are different predictors of complications in each subgroup, overall SFS, complications and LOH are similar. We observed that PCNL is safe in children and the geriatric population with SFS and complications similar to adults.

Being a retrospective study, we have taken adequate steps to avoid the inherent problems of this study design. However, it is possible that some bias could still exist. We have excluded patients with incomplete data. We used only XRKUB and US for assessing residual fragments. We could not perform CT KUB in every patient due to the additional costs involved. We hence defined stone free status as absence of any residual fragment >4mm. Our data includes complications of prone PCNL from a single center. They may need external validation.

## CONCLUSIONS

Complications in prone PCNL are similar across pediatric, adult and geriatric subgroups. Age does not alter the incidence of complications or the grade of complications, stone free status and length of hospital stay, contrary to the belief that these patient populations are unique due to extremes of age and associated comorbidities. Need for nephrostomy in children and prolonged operation duration in geriatric patients were independent predictors of complications. In adults, supracostal access, complex renal stones, need of nephrostomy and prolonged operation duration predicted a higher complication rate.
